# Supplementary material for: A haplotype-resolved view of human gene regulation
Source: bioRxiv. 2025 Jun 2:2024.06.14.599122. Preprint. [Version 2] doi: 10.1101/2024.06.14.599122 (PMC12157683; doi:10.1101/2024.06.14.599122)
Supplement: Supplement 2 — Figure S1. Motivation for the FIRE model: heterogeneity in single-molecule data. a) Range of m6A concentrations in Fiber-seq experiments used in training of the FIRE model. b) Heterogeneity of m6A concentrations between reads in the Fiber-seq experiments used in the training of the FIRE model. c). Distribution of the length of methyltransferase-sensitive patches (MSPs) across the Fiber-seq experiments used in training the FIRE model. d) Number of actuated regulatory elements within DNase I hypersensitive sites (DHSs) on chromosome 20 (y-axis) vs the number of actuated regulatory elements outside of DHSs for different FIRE (red) and MSP length (gray) thresholds (x-axis). e) Distribution of MSP length within DNase peaks and outside of DNase peaks (grey), stratified by inferred FIRE elements (red) and nucleosomal linker regions (purple). Figure S2. Training of the FIRE model. a) Schematic of training Fiber-seq inferred regulatory elements (FIREs) using XGBoost within the Mokapot framework. b) Schematic of windows along single reads used for calculating features in the FIRE model. Descriptions of each feature are listed in Table S3. C) Ranking the importance of features in the XGBoost model using the feature score (F score), which sums up how many times each feature is split on within the model. Figure S3. FIRE elements and their underlying m6A calls. UCSC genome browser screenshots of a low accessibility regulatory element (a), a high accessibility element (b), and two sites without regulatory elements (c,d). Shown in each panel in order are: the gene models, ATAC signal, percent of Fiber-seq reads with FIRE elements, wide and narrow FIRE peak calls, raw m6A calls for individual Fiber-seq reads, and FIRE calls for individual Fiber-seq reads in the same order. Figure S4. The aggregate FIRE score and peak calling with the FIRE method. a) Schematic of calculating a false discovery rate (FDR) for the aggregated FIRE score. top) the aggregated FIRE score across multiple Fib [file media-2.pdf]

Figure S1

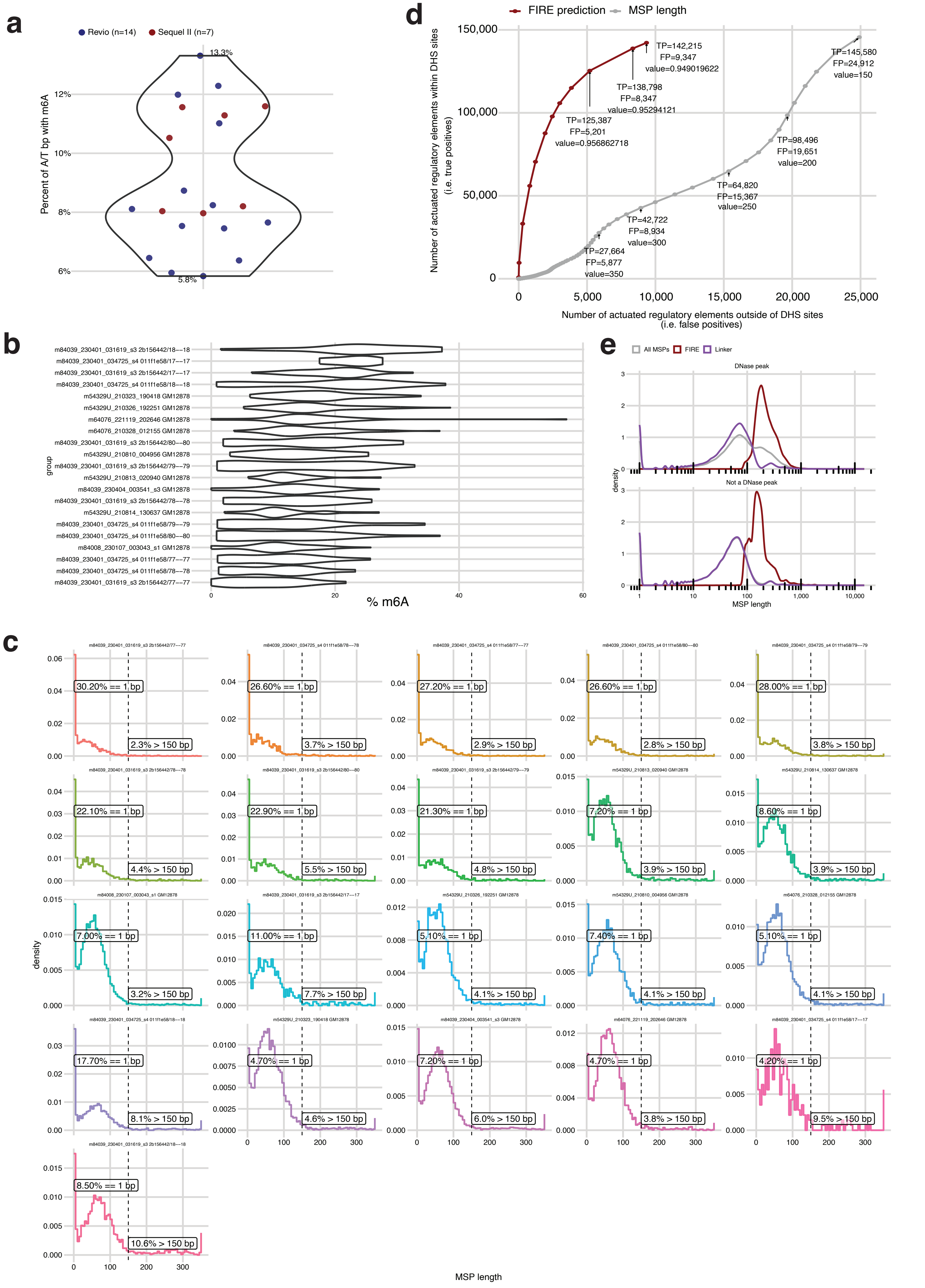
a
Revio (n=14)
Sequel II (n=7)
Percent of A/T bp with m6A
13.3%
5.8%
12%
10%
8%
6%
d
FIRE prediction
MSP length
Number of actuated regulatory elements within DHS sites
(i.e. true positives)
Number of actuated regulatory elements outside of DHS sites
(i.e. false positives)
TP=142,215
FP=9,347
value=0.949019622
TP=138,798
FP=8,347
value=0.95294121
TP=125,387
FP=5,201
value=0.956862718
TP=145,580
FP=24,912
value=150
TP=98,496
FP=19,651
value=200
TP=64,820
FP=15,367
value=250
TP=42,722
FP=8,934
value=300
TP=27,664
FP=5,877
value=350
b
group
% m6A
e
All MSPs
FIRE
Linker
DNase peak
Not a DNase peak
density
MSP length
c
density
MSP length

Figure S2

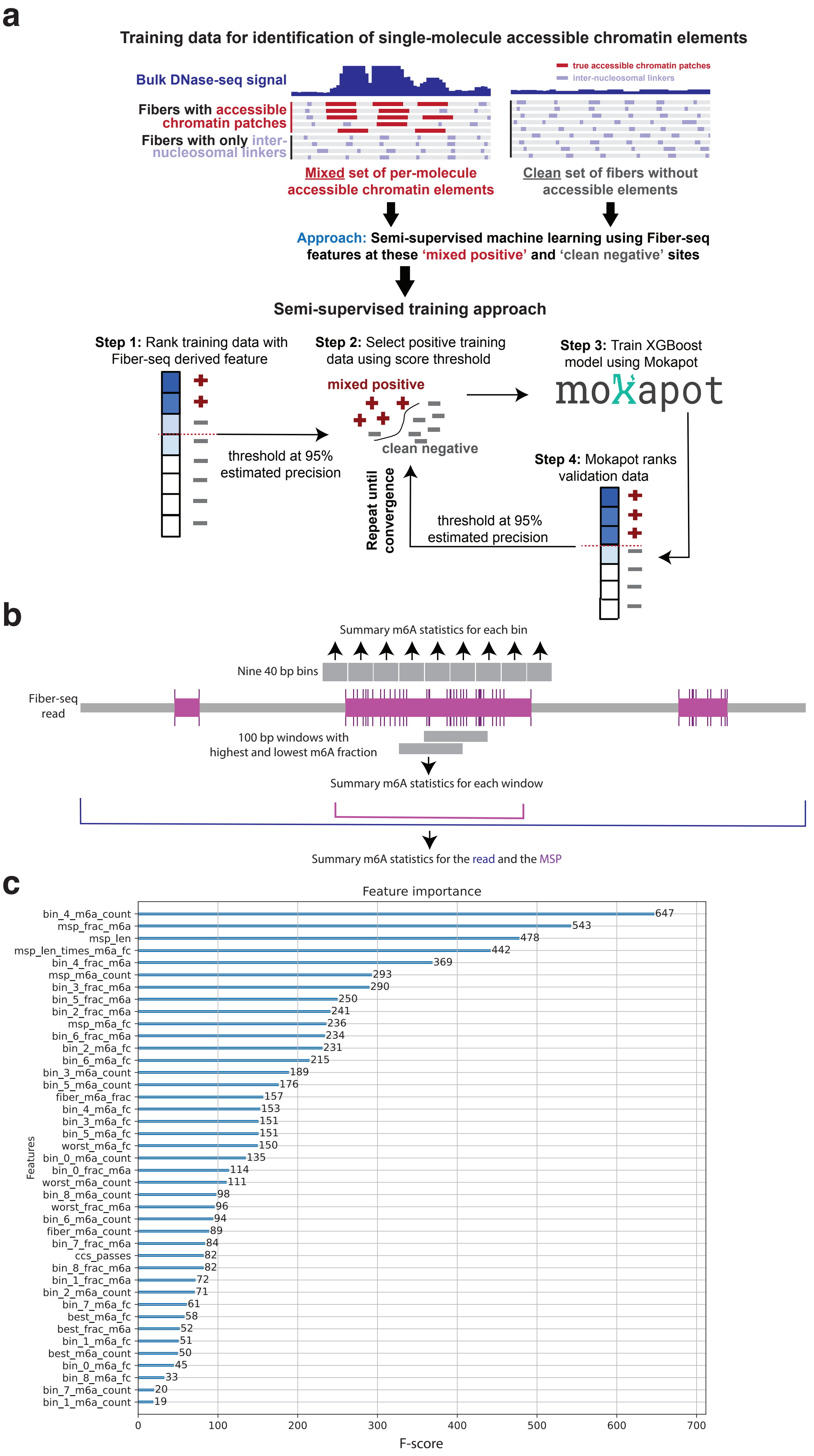
a
Training data for identification of single-molecule accessible chromatin elements
Bulk DNase-seq signal
true accessible chromatin patches
inter-nucleosomal linkers
Fibers with accessible chromatin patches
Fibers with only inter-nucleosomal linkers
Mixed set of per-molecule accessible chromatin elements
Clean set of fibers without accessible elements
Approach: Semi-supervised machine learning using Fiber-seq features at these 'mixed positive' and 'clean negative' sites
Semi-supervised training approach
Step 1: Rank training data with Fiber-seq derived feature
Step 2: Select positive training data using score threshold
Step 3: Train XGBoost model using Mokapot
mokapot
mixed positive
clean negative
threshold at 95% estimated precision
Repeat until convergence
Step 4: Mokapot ranks validation data
threshold at 95% estimated precision
b
Summary m6A statistics for each bin
Nine 40 bp bins
Fiber-seq read
100 bp windows with highest and lowest m6A fraction
Summary m6A statistics for each window
Summary m6A statistics for the read and the MSP
c
Feature importance
Features
F-score
bin_4_m6a_count 647
msp_frac_m6a 543
msp_len 478
msp_len_times_m6a_fc 442
bin_4_frac_m6a 369
msp_m6a_count 293
bin_3_frac_m6a 290
bin_5_frac_m6a 250
bin_2_frac_m6a 241
msp_m6a_fc 236
bin_6_frac_m6a 234
bin_2_m6a_fc 231
bin_6_m6a_fc 215
bin_3_m6a_count 189
bin_5_m6a_count 176
fiber_m6a_frac 157
bin_4_m6a_fc 153
bin_3_m6a_fc 151
bin_5_m6a_fc 151
worst_m6a_fc 150
bin_0_m6a_count 135
bin_0_frac_m6a 114
worst_m6a_count 111
bin_8_m6a_count 98
worst_frac_m6a 96
bin_6_m6a_count 94
fiber_m6a_count 89
bin_7_frac_m6a 84
ccs_passes 82
bin_8_frac_m6a 82
bin_1_frac_m6a 72
bin_2_m6a_count 71
bin_7_m6a_fc 61
best_m6a_fc 58
best_frac_m6a 52
bin_1_m6a_fc 51
best_m6a_count 50
bin_0_m6a_fc 45
bin_8_m6a_fc 33
bin_7_m6a_count 20
bin_1_m6a_count 19
0 100 200 300 400 500 600 700

Figure S3

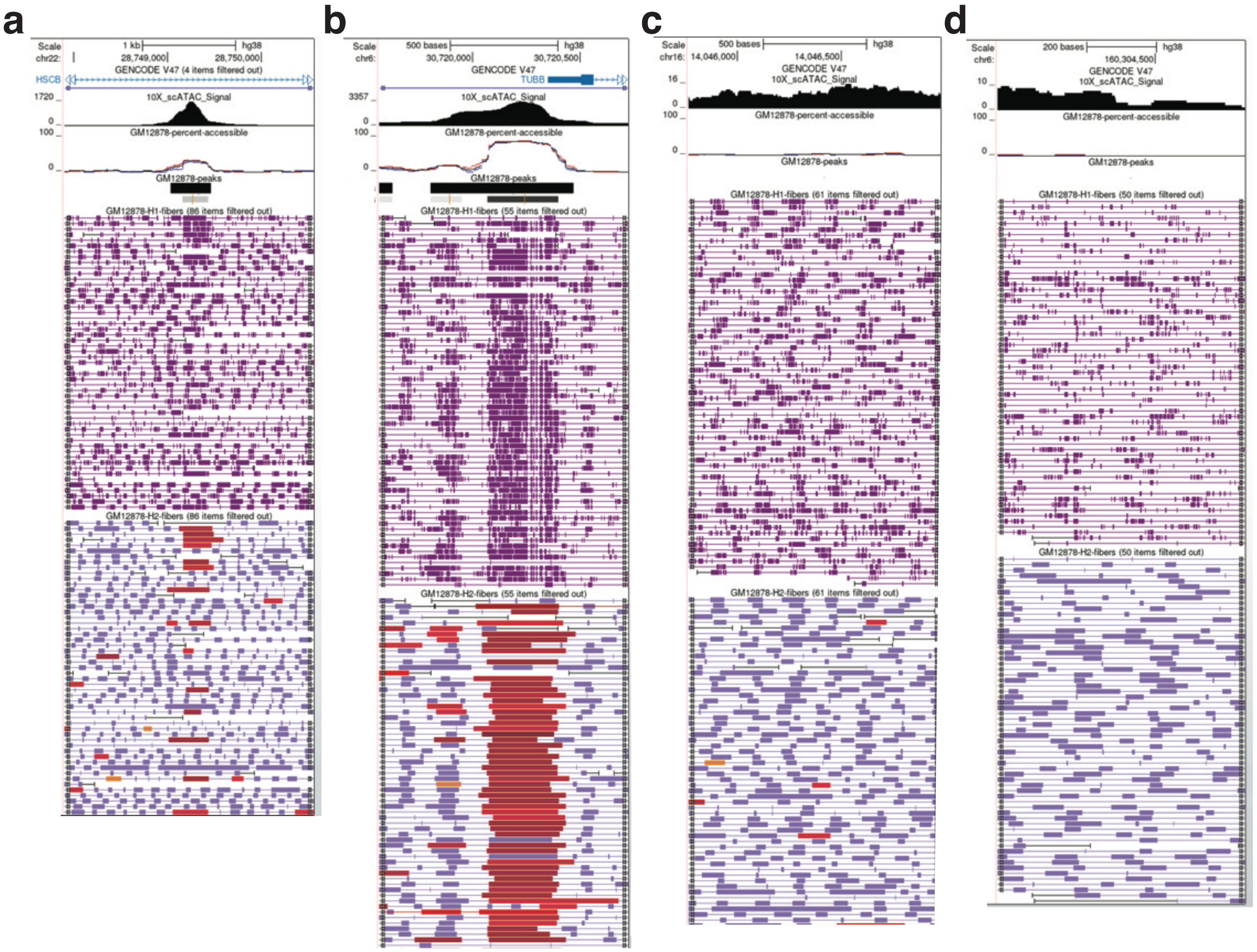

a
Scale
chr22:
1 kb
hg38
28,749,000
28,750,000
GENCODE V47 (4 items filtered out)
HSCB
1720
10X_scATAC_Signal
0
100
GM12878-percent-accessible
0
GM12878-peaks
GM12878-H1-fibers (86 items filtered out)
GM12878-H2-fibers (86 items filtered out)
b
Scale
chr6:
500 bases
hg38
30,720,000
30,720,500
GENCODE V47
TUBB
3357
10X_scATAC_Signal
0
100
GM12878-percent-accessible
0
GM12878-peaks
GM12878-H1-fibers (55 items filtered out)
GM12878-H2-fibers (55 items filtered out)
c
Scale
chr16:
500 bases
hg38
14,046,000
14,046,500
GENCODE V47
10X_scATAC_Signal
16
0
100
GM12878-percent-accessible
0
GM12878-peaks
GM12878-H1-fibers (61 items filtered out)
GM12878-H2-fibers (61 items filtered out)
d
Scale
chr6:
200 bases
hg38
160,304,500
GENCODE V47
10X_scATAC_Signal
10
0
100
GM12878-percent-accessible
0
GM12878-peaks
GM12878-H1-fibers (50 items filtered out)
GM12878-H2-fibers (50 items filtered out)

Figure S4

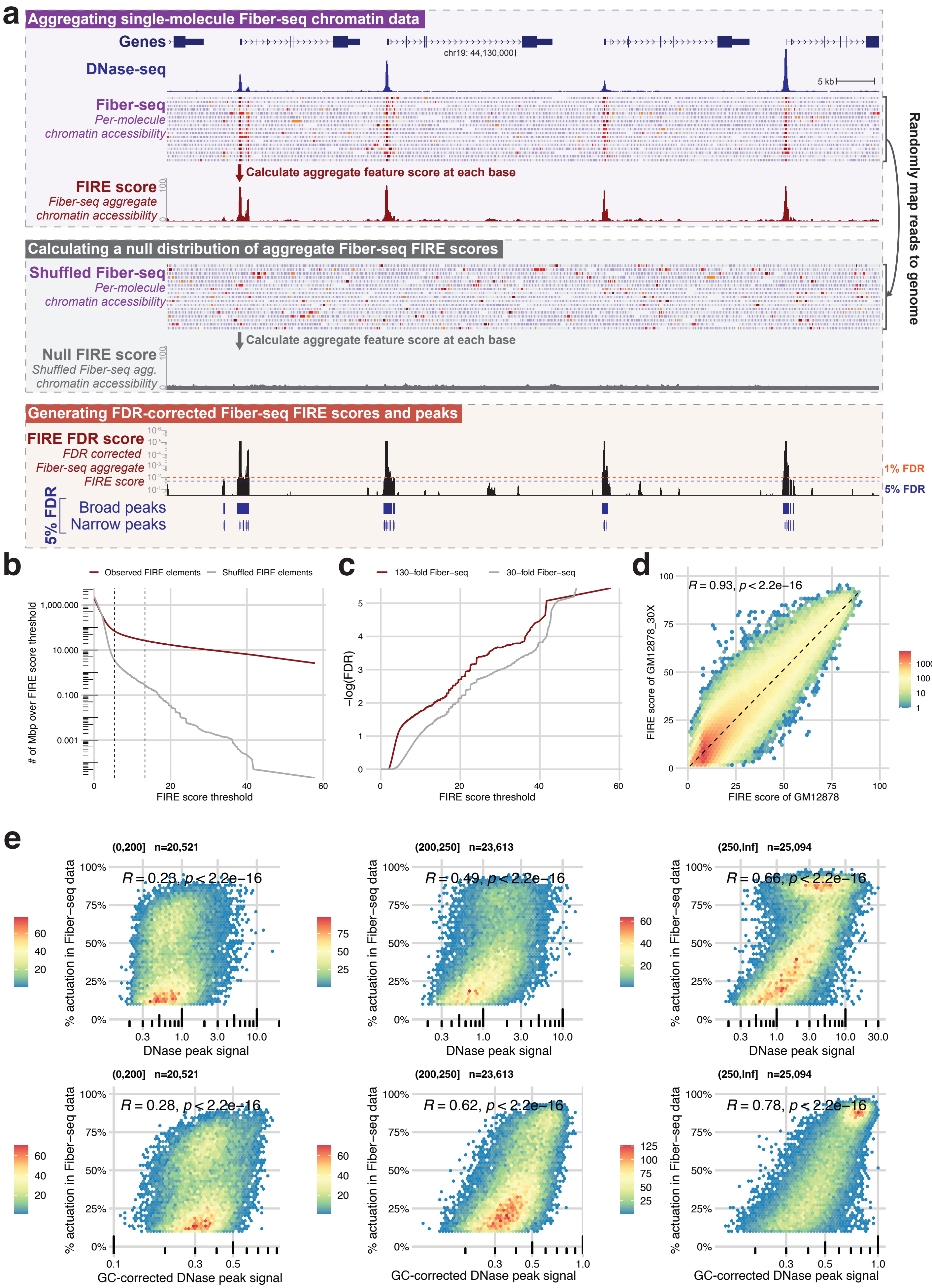

a
Aggregating single-molecule Fiber-seq chromatin data
Genes
chr19: 44,130,000
DNase-seq
5 kb
Fiber-seq
Per-molecule chromatin accessibility
Calculate aggregate feature score at each base
FIRE score
Fiber-seq aggregate chromatin accessibility
Randomly map reads to genome
Calculating a null distribution of aggregate Fiber-seq FIRE scores
Shuffled Fiber-seq
Per-molecule chromatin accessibility
Calculate aggregate feature score at each base
Null FIRE score
Shuffled Fiber-seq agg. chromatin accessibility
Generating FDR-corrected Fiber-seq FIRE scores and peaks
FIRE FDR score
FDR corrected Fiber-seq aggregate FIRE score
1% FDR
5% FDR
Broad peaks
Narrow peaks
b
Observed FIRE elements
Shuffled FIRE elements
# of Mbp over FIRE score threshold
FIRE score threshold
c
130−fold Fiber−seq
30−fold Fiber−seq
−log(FDR)
FIRE score threshold
d
R = 0.93, p < 2.2e−16
FIRE score of GM12878_30X
FIRE score of GM12878
e
(0,200] n=20,521
R = 0.23, p < 2.2e−16
(200,250] n=23,613
R = 0.49, p < 2.2e−16
(250,Inf] n=25,094
R = 0.66, p < 2.2e−16
% actuation in Fiber−seq data
DNase peak signal
(0,200] n=20,521
R = 0.28, p < 2.2e−16
(200,250] n=23,613
R = 0.62, p < 2.2e−16
(250,Inf] n=25,094
R = 0.78, p < 2.2e−16
GC-corrected DNase peak signal

Figure S5

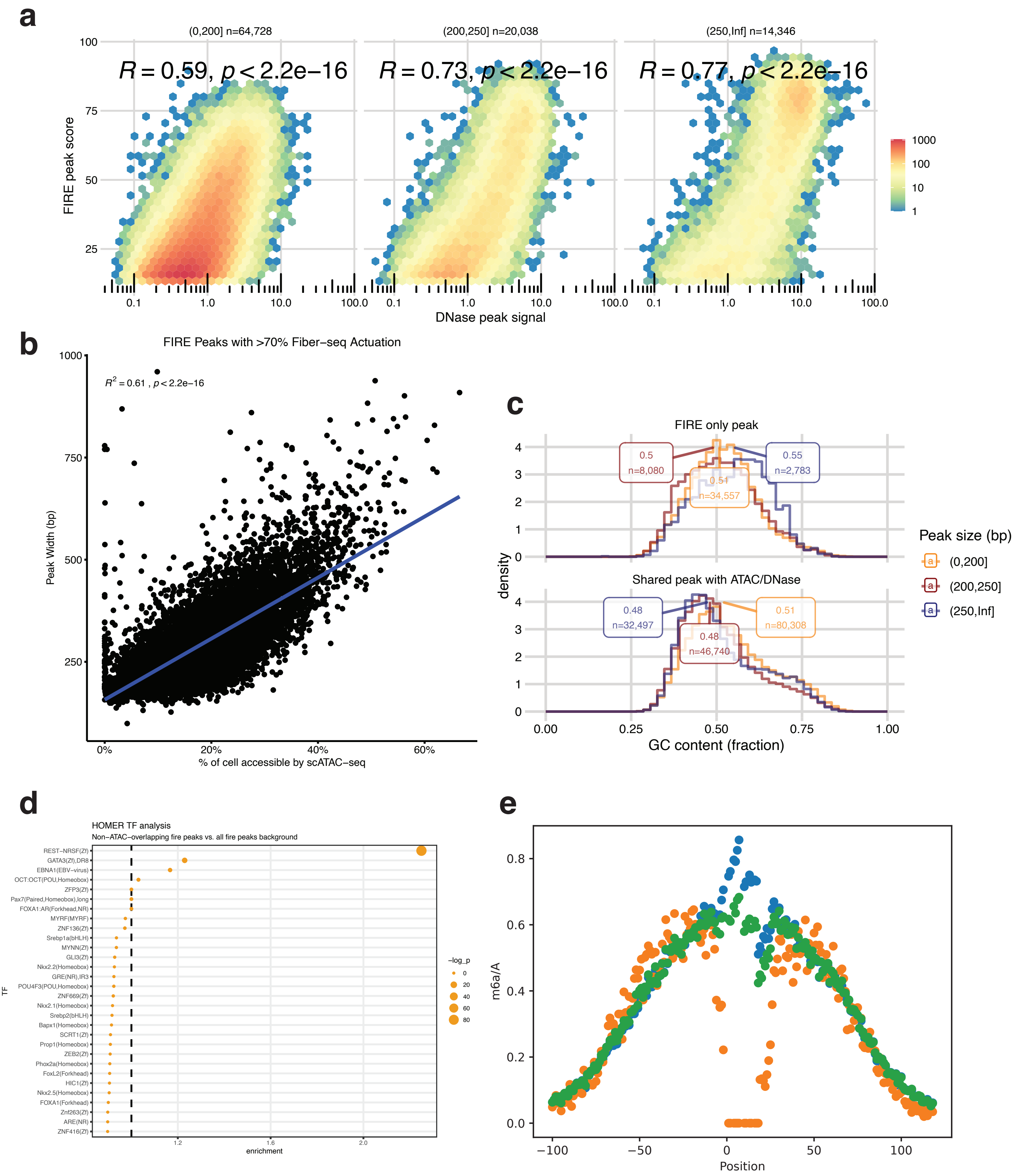
a
(0,200] n=64,728
(200,250] n=20,038
(250,Inf] n=14,346
R = 0.59, p < 2.2e−16
R = 0.73, p < 2.2e−16
R = 0.77, p < 2.2e−16
FIRE peak score
DNase peak signal
1000
100
10
1
b
FIRE Peaks with >70% Fiber−seq Actuation
R² = 0.61 , p < 2.2e−16
Peak Width (bp)
% of cell accessible by scATAC−seq
c
FIRE only peak
Shared peak with ATAC/DNase
0.5
n=8,080
0.55
n=2,783
0.51
n=34,557
0.48
n=32,497
0.51
n=80,308
0.48
n=46,740
density
GC content (fraction)
Peak size (bp)
(0,200]
(200,250]
(250,Inf]
d
HOMER TF analysis
Non−ATAC−overlapping fire peaks vs. all fire peaks background
REST−NRSF(Zf)
GATA3(Zf),DR8
EBNA1(EBV−virus)
OCT:OCT(POU,Homeobox)
ZFP3(Zf)
Pax7(Paired,Homeobox),long
FOXA1:AR(Forkhead,NR)
MYRF(MYRF)
ZNF136(Zf)
Srebp1a(bHLH)
MYNN(Zf)
GLI3(Zf)
Nkx2.2(Homeobox)
GRE(NR),IR3
POU4F3(POU,Homeobox)
ZNF669(Zf)
Nkx2.1(Homeobox)
Srebp2(bHLH)
Bapx1(Homeobox)
SCRT1(Zf)
Prop1(Homeobox)
ZEB2(Zf)
Phox2a(Homeobox)
FoxL2(Forkhead)
HIC1(Zf)
Nkx2.5(Homeobox)
FOXA1(Forkhead)
Znf263(Zf)
ARE(NR)
ZNF416(Zf)
TF
enrichment
−log_p
e
m6a/A
Position

Figure S6

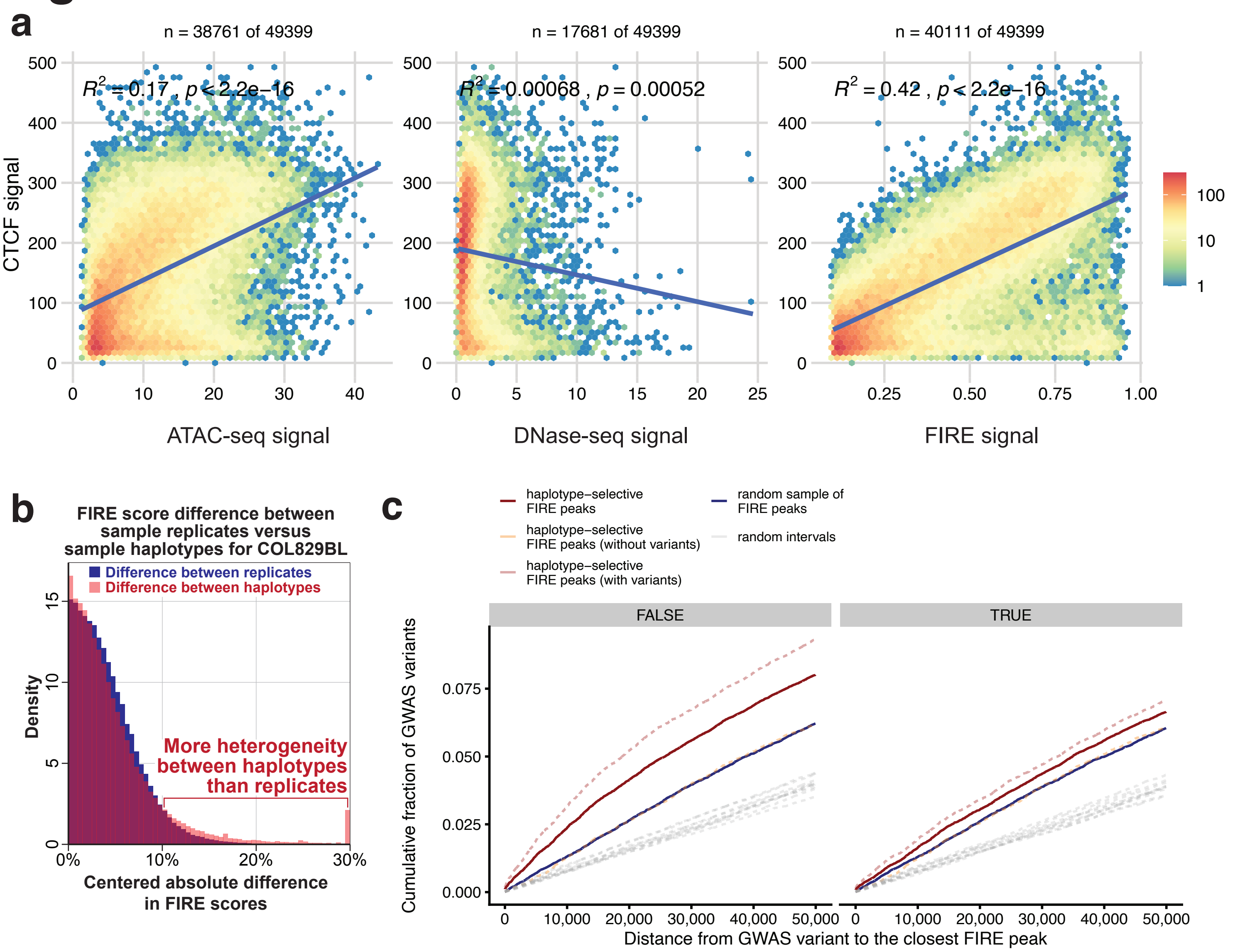
a
n = 38761 of 49399
n = 17681 of 49399
n = 40111 of 49399
$R^2 = 0.17$, $p < 2.2e-16$
$R^2 = 0.00068$, $p = 0.00052$
$R^2 = 0.42$, $p < 2.2e-16$
CTCF signal
ATAC-seq signal
DNase-seq signal
FIRE signal
100
10
1
b
FIRE score difference between sample replicates versus sample haplotypes for COL829BL
Difference between replicates
Difference between haplotypes
More heterogeneity between haplotypes than replicates
Density
Centered absolute difference in FIRE scores
c
haplotype−selective FIRE peaks
haplotype−selective FIRE peaks (without variants)
haplotype−selective FIRE peaks (with variants)
random sample of FIRE peaks
random intervals
FALSE
TRUE
Cumulative fraction of GWAS variants
Distance from GWAS variant to the closest FIRE peak

Figure S7

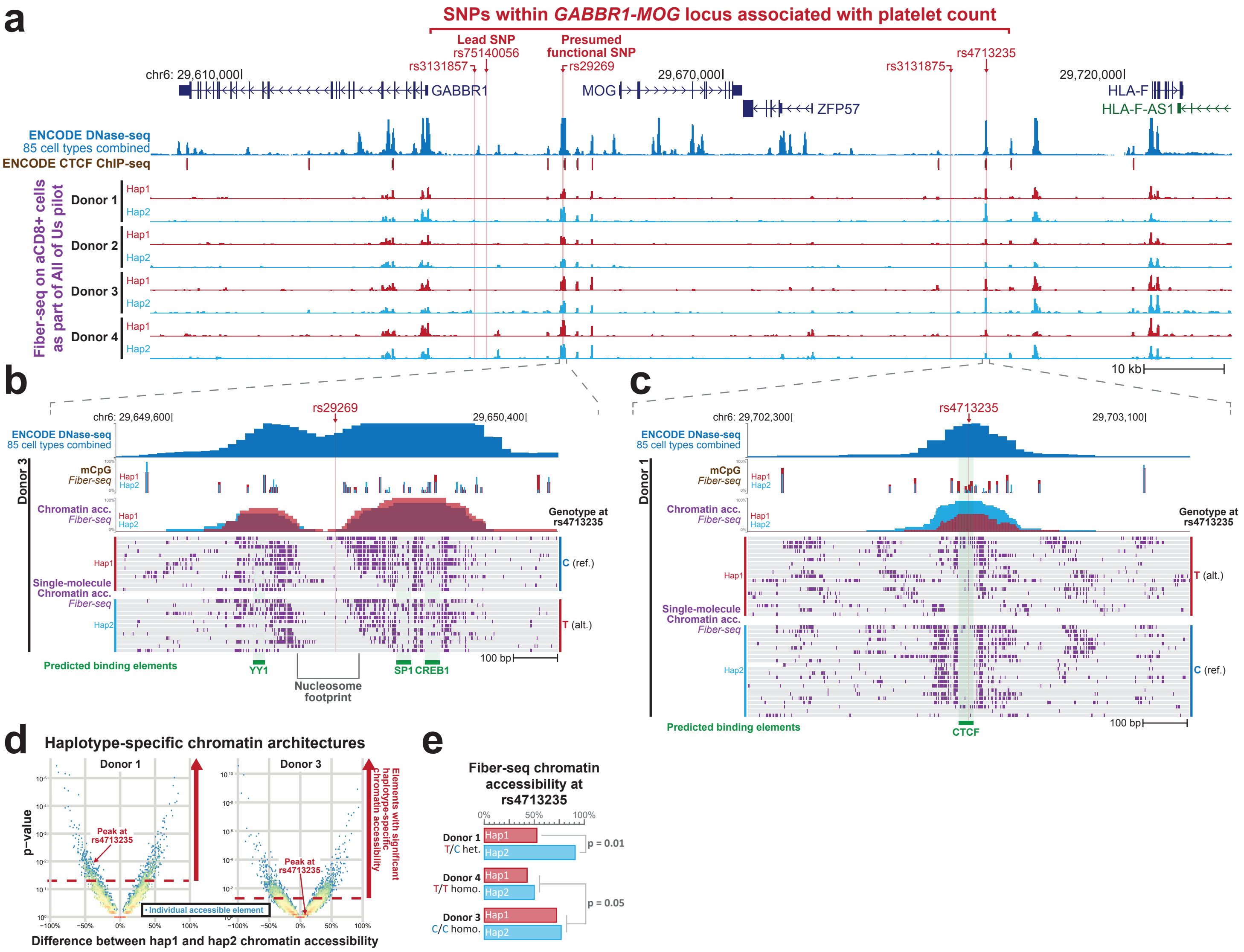

a
SNPs within GABBR1-MOG locus associated with platelet count
Lead SNP
rs75140056
Presumed functional SNP
rs3131857
rs29269
rs3131875
rs4713235
chr6: 29,610,000
29,670,000
29,720,000
GABBR1
MOG
ZFP57
HLA-F
HLA-F-AS1
ENCODE DNase-seq
85 cell types combined
ENCODE CTCF ChIP-seq
Fiber-seq on aCD8+ cells as part of All of Us pilot
Donor 1
Donor 2
Donor 3
Donor 4
Hap1
Hap2
10 kb
b
chr6: 29,649,600
29,650,400
mCpG
Fiber-seq
Chromatin acc.
Single-molecule Chromatin acc.
Genotype at rs4713235
C (ref.)
T (alt.)
Predicted binding elements
YY1
Nucleosome footprint
SP1
CREB1
100 bp
c
chr6: 29,702,300
29,703,100
CTCF
d
Haplotype-specific chromatin architectures
p-value
Peak at rs4713235
Individual accessible element
Elements with significant haplotype-specific chromatin accessibility
Difference between hap1 and hap2 chromatin accessibility
e
Fiber-seq chromatin accessibility at rs4713235
Donor 1 T/C het.
Donor 4 T/T homo.
Donor 3 C/C homo.
p = 0.01
p = 0.05

Figure S8

a

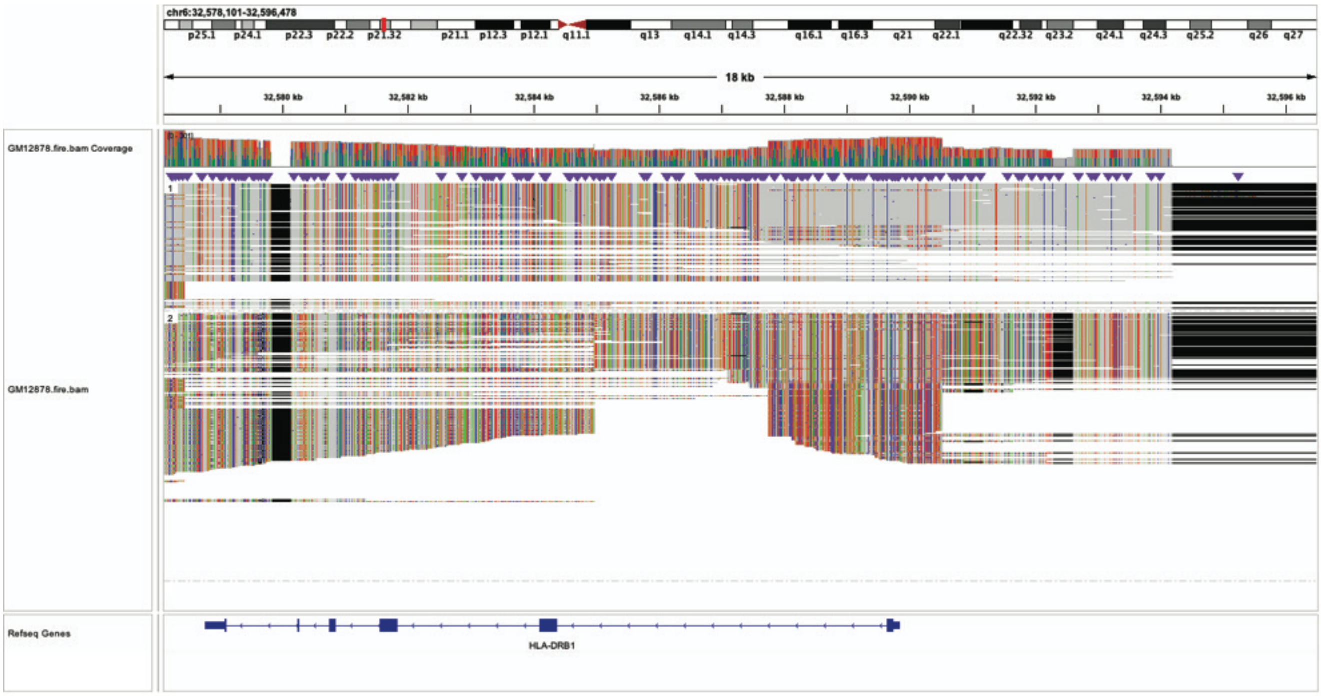

b

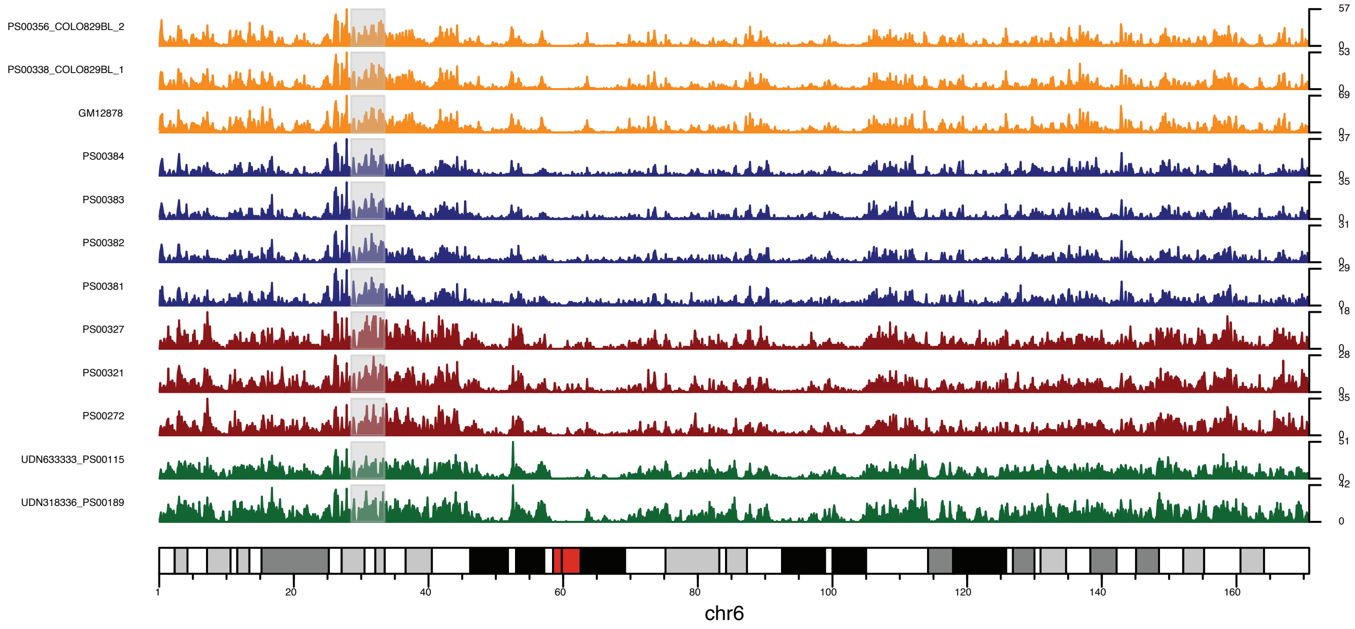

c

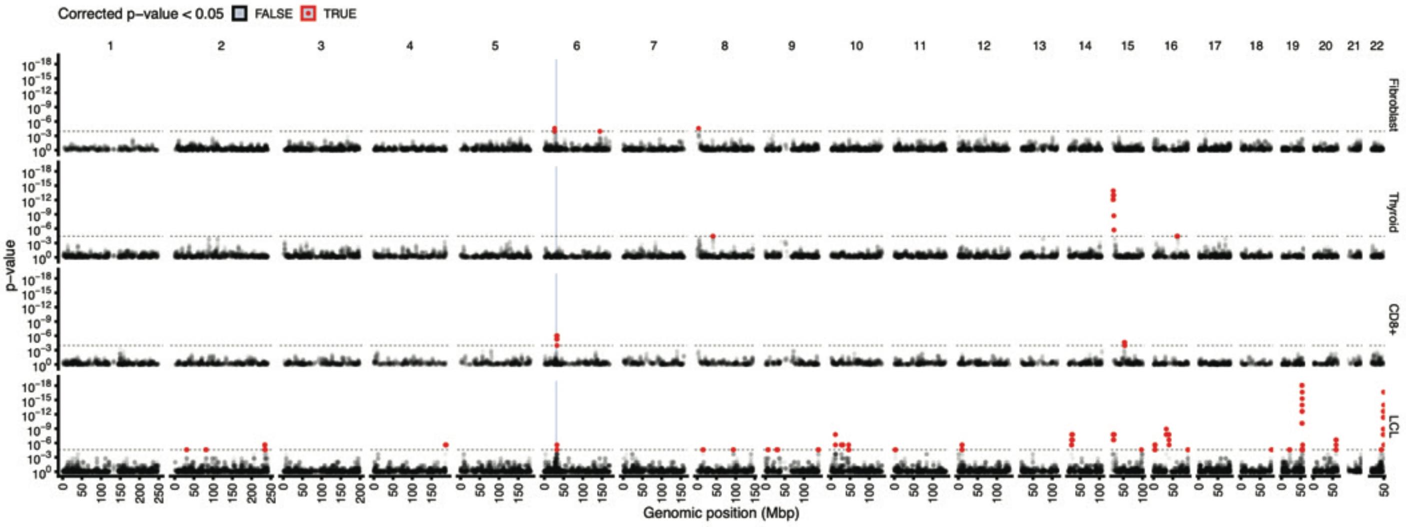

Figure S9

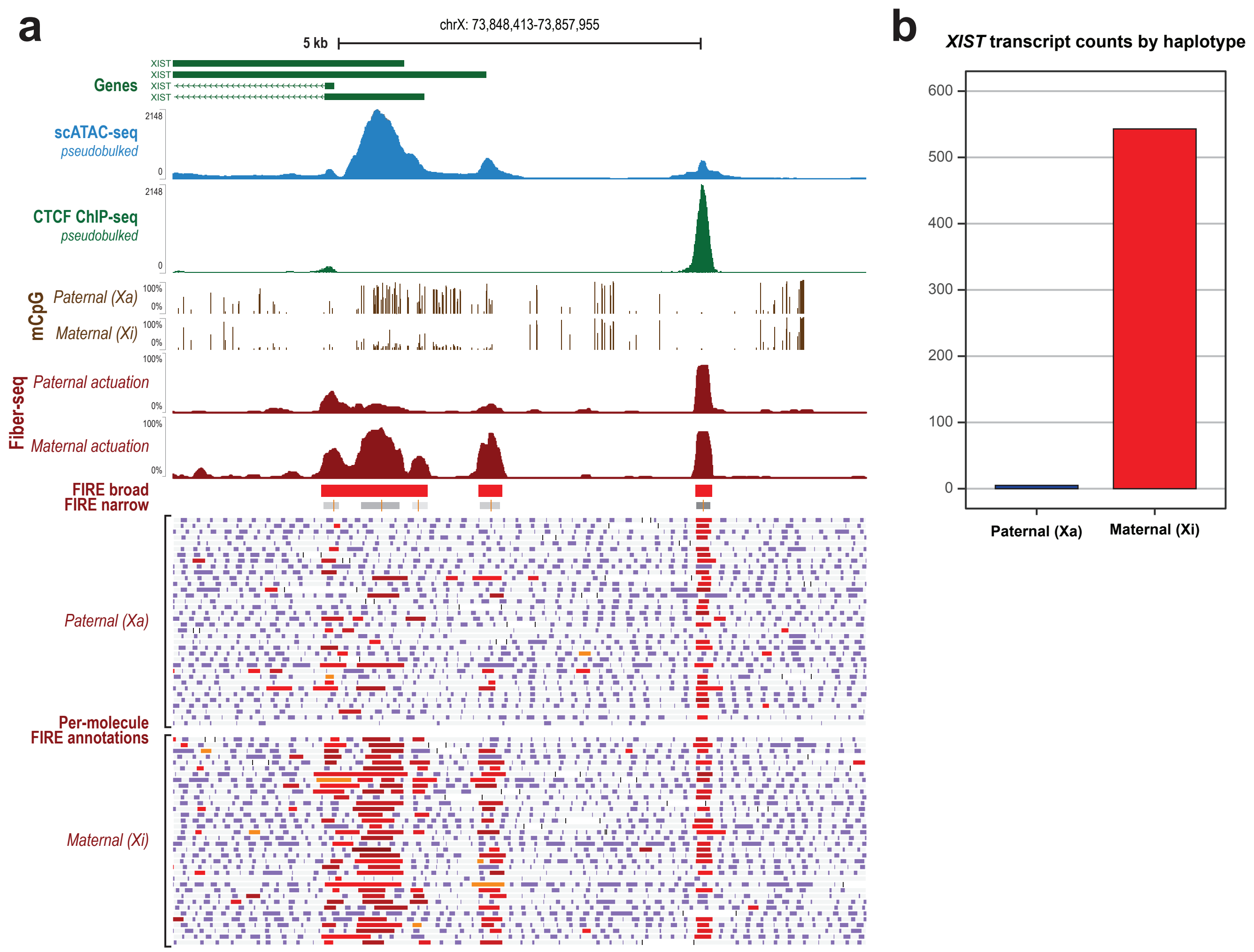
a
chrX: 73,848,413-73,857,955
5 kb
XIST
XIST
XIST
XIST
Genes
scATAC-seq
pseudobulked
2148
0
CTCF ChIP-seq
pseudobulked
2148
0
mCpG
Paternal (Xa)
100%
0%
Maternal (Xi)
100%
0%
Fiber-seq
Paternal actuation
100%
0%
Maternal actuation
100%
0%
FIRE broad
FIRE narrow
Paternal (Xa)
Per-molecule
FIRE annotations
Maternal (Xi)
b
XIST transcript counts by haplotype
600
500
400
300
200
100
0
Paternal (Xa)
Maternal (Xi)

Figure S10

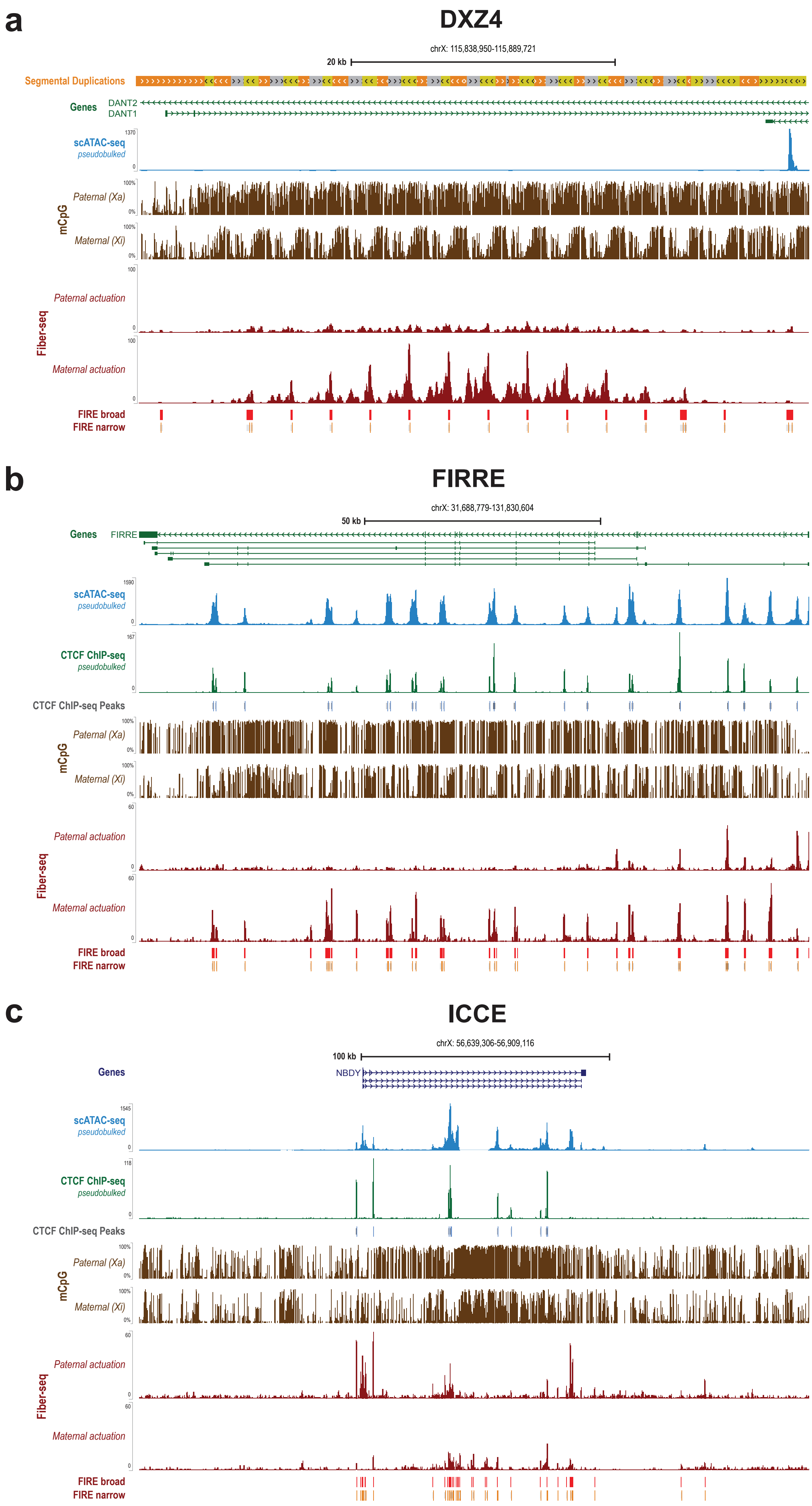

a
DXZ4
chrX: 115,838,950-115,889,721
20 kb
Segmental Duplications
Genes
DANT2
DANT1
scATAC-seq
pseudobulked
1370
0
mCpG
Paternal (Xa)
Maternal (Xi)
100%
0%
Fiber-seq
Paternal actuation
Maternal actuation
100
0
FIRE broad
FIRE narrow
b
FIRRE
chrX: 31,688,779-131,830,604
50 kb
Genes
FIRRE
scATAC-seq
pseudobulked
1590
CTCF ChIP-seq
pseudobulked
167
CTCF ChIP-seq Peaks
mCpG
Paternal (Xa)
Maternal (Xi)
Fiber-seq
Paternal actuation
Maternal actuation
60
FIRE broad
FIRE narrow
c
ICCE
chrX: 56,639,306-56,909,116
100 kb
Genes
NBDY
scATAC-seq
pseudobulked
1545
CTCF ChIP-seq
pseudobulked
118
CTCF ChIP-seq Peaks
mCpG
Paternal (Xa)
Maternal (Xi)
Fiber-seq
Paternal actuation
Maternal actuation
60
FIRE broad
FIRE narrow
